# Radiomic Profiling of Head and Neck Cancer: ^18^F-FDG PET Texture Analysis as Predictor of Patient Survival

**DOI:** 10.1155/2018/3574310

**Published:** 2018-09-27

**Authors:** G. Feliciani, F. Fioroni, E. Grassi, M. Bertolini, A. Rosca, G. Timon, M. Galaverni, C. Iotti, A. Versari, M. Iori, P. Ciammella

**Affiliations:** ^1^Medical Physics Unit, Istituto Scientifico Romagnolo per lo Studio e la Cura dei Tumori (IRST) IRCCS, Meldola, Italy; ^2^Medical Physics Unit, AUSL-IRCCS, Reggio Emilia, Italy; ^3^Radiation Oncology Unit, AUSL-IRCCS, Reggio Emilia, Italy; ^4^Nuclear Medicine Unit, AUSL-IRCCS, Reggio Emilia, Italy

## Abstract

**Background and Purpose:**

The accurate prediction of prognosis and pattern of failure is crucial for optimizing treatment strategies for patients with cancer, and early evidence suggests that image texture analysis has great potential in predicting outcome both in terms of local control and treatment toxicity. The aim of this study was to assess the value of pretreatment ^18^F-FDG PET texture analysis for the prediction of treatment failure in primary head and neck squamous cell carcinoma (HNSCC) treated with concurrent chemoradiation therapy.

**Methods:**

We performed a retrospective analysis of 90 patients diagnosed with primary HNSCC treated between January 2010 and June 2017 with concurrent chemo-radiotherapy. All patients underwent ^18^F-FDG PET/CT before treatment. ^18^F-FDG PET/CT texture features of the whole primary tumor were measured using an open-source texture analysis package. Least absolute shrinkage and selection operator (LASSO) was employed to select the features that are associated the most with clinical outcome, as progression-free survival and overall survival. We performed a univariate and multivariate analysis between all the relevant texture parameters and local failure, adjusting for age, sex, smoking, primary tumor site, and primary tumor stage. Harrell *c*-index was employed to score the predictive power of the multivariate cox regression models.

**Results:**

Twenty patients (22.2%) developed local failure, whereas the remaining 70 (77.8%) achieved durable local control. Multivariate analysis revealed that one feature, defined as low-intensity long-run emphasis (LILRE), was a significant predictor of outcome regardless of clinical variables (hazard ratio < 0.001, *P*=0.001).The multivariate model based on imaging biomarkers resulted superior in predicting local failure with a *c*-index of 0.76 against 0.65 of the model based on clinical variables alone.

**Conclusion:**

LILRE, evaluated on pretreatment ^18^F-FDG PET/CT, is associated with higher local failure in patients with HNSCC treated with chemoradiotherapy. Using texture analysis in addition to clinical variables may be useful in predicting local control.

## 1. Introduction

Concurrent chemoradiotherapy (CRT) is the mainstay of treatment for early and locally advanced head and neck squamous cell carcinoma (HNSCC) [1, 2]. The accurate prediction of prognosis and failure in these patients is crucial for optimizing treatment. Some clinical features are commonly accepted as risk factors, such as tumor size, local anatomic invasion, nodal involvement, presence of distant metastases, and HPV status [3–5].

Nowadays, imaging plays a central role in the investigation of tumor prognosis. Radiological images are acquired as routine practice for almost every patient with HNSCC and represent an immense source of potential data for decoding tumor phenotypes and tumor heterogeneity [6–10]. Image texture is defined as a complex visual pattern within an image, consisting of simpler subpatterns with characteristic features, and texture analysis allows the mathematic detection of tumor heterogeneity. In the past years, CT texture analysis has been investigated in oncologic imaging for its ability to predict treatment outcome in patients with various tumors (lung, esophageal, and renal cancer) [11–13]. New and more descriptive metrics of tumor heterogeneity based on texture analysis (TA) of ^18^F-FDG PET images are now used to further improve outcome prediction [14–17] in different types of cancer [18, 19], including HNSCC.

Despite the promising results achieved in preliminary studies, TA still suffers from standardization issues, and the scientific community agreement is missing [20, 21]. The absolute values of the TA features used for stratifying patients depend on a number of variables, starting from image acquisition parameters to the contouring algorithm employed for tumor segmentation. Previous studies on phantoms and patients exploited these dependencies trying to find a common way to apply TA and standardize results [20, 22–25]. In the present study, we retrospectively apply TA to ^18^F-FDG PET/CT images of 90 patients affected by HNSCC and treated with CRT in order to identify imaging biomarkers able to predict patients' outcomes such as overall survival (OS) and progression-free survival (PFS). We compared TA features prognostic values with standard clinical variables such as age, sex, size and site of primary tumors, clinical stage, and nodal involvement.

## 2. Materials and Methods

### 2.1. Patients

We enrolled 129 patients (median age 60 years, range 22–87 years) with diagnosis of HNSCC consecutively treated between January 2010 and June 2017 with concurrent CRT with curative intent at the Radiation Oncology Unit of our institution. The ethics committee of our hospital approved this retrospective study, and informed consent was obtained from all participants. All patients underwent ^18^F-FDG PET/CT before treatment for initial staging and were followed up for at least 5 years after treatment or until death. Patients' medical records were retrospectively analyzed to extract information for outcome assessment such as details about therapy, last follow up date, disease status, pattern of recurrence, death and cause of death. Patients' demographics and clinical characteristics, including age, sex, site of primary tumor, clinical stage, and type of treatment and are summarized in Table 1. Tumor sites were oral cavity, larynx, oropharynx, and hypopharynx in 5%, 20%, 60%, and 15% of patients, respectively.

### 2.2. Treatment Regimens and Follow-Up

All patients were uniformly treated with intensity-modulated radiation therapy (IMRT) and concurrent platinum-based chemotherapy. Weekly cisplatin was the primary choice of chemotherapeutic agent. Forty-three patients received neoadjuvant chemotherapy before CRT. The decision regarding the use of adjuvant chemotherapy was individualized, based on the extent of disease, medical conditions of the patient, and the radiation oncologist opinion. Most of the patients were treated with a simultaneous integrated boost (SIB) technique, with a total dose on gross tumor volume up to 66 Gy or equivalent in 30–33 fractions (mainly 66 Gy/2.2 Gy per fraction); the high-intermediate risk cervical lymphatic areas received 54–60 Gy or equivalent. Patients were followed after the conclusion of treatment to evaluate local control. All patients were followed clinically for at least 5 years after completion of CRT, every 1–3 months for the first two years, every 4–6 months for the next three years, and annually thereafter.

The follow-up evaluation included physical and endoscopic examinations jointly performed by radiation oncologist and otorhinolaryngologist. In addition, CT, MRI, and ^18^F-FDG PET/CT imaging were used to assess the clinical response and were usually performed within 6 months from the end of the treatment. Recurrence or distant metastasis were diagnosed based on either a positive biopsy or clinical/radiographic evidence of progression. The follow-up period was designated as the total time of follow-up, starting at treatment initiation and ending either at histologically confirmed local failure, or at radiologically systemic recurrence, or at last failure-free patient contact.

### 2.3. ^18^F-FDG PET/CT Imaging Protocol

Patients underwent imaging on a GE Discovery STE16 PET/CT scanner before treatment using a standard PET/CT clinical protocol. Patients fasted for at least 6 h before the intravenous administration of ^18^F-FDG (3.7 MBq/kg). Serum glucose concentrations were measured before FDG injection and were less than 150 mg/dl, or if between 150 and 200 mg/dl, the patient was hydrated and glucose concentration measured after 40–50 min. The scans were acquired with the patients immobilized in radiation treatment position using a dedicated flat table and personalized thermoplastic masks. PET images were corrected for random and scatter noise components and then reconstructed on a 256 × 256 image matrix using 3-D VUE Point HD algorithm (two iterations, 28 subsets, postfilter 6 mm) corrected for attenuation. Pixel spacing was of 2.73 × 2.73 mm with 3.27 mm slice thickness.

The qualitative and quantitative PET imaging evaluations were performed for each patient by two-blinded expert nuclear medicine physicians using the PET/CT fused images in transverse, coronal, and sagittal planes. A consensus was then reached by comparison of the two evaluations. The presence of abnormal FDG uptake, excluding the areas of physiologically increased uptake, was considered suspicious for malignancy. The readers had knowledge of all available clinical and imaging information related to the patients.

An expert nuclear medicine physician together with a medical physicist and a radiation oncologist, using a semiautomatic segmentation technique, outlined the primary tumor. The image segmentation of the whole primary lesion for each section was performed with a dedicated Advantage Workstation v 4.4 (GE Medical System, USA), using the 40% SUV_max_ isocontour algorithm to avoid operator dependence in contouring and any other manual adjustments. We assumed 40% threshold to acquire standardization with other works that perform prediction analysis [15]. Contours were then checked by visual inspection.

### 2.4. Texture Analysis

We extracted 75 features from each segmented tissue volume using an Open-Source Package “CGITA” version 1.3 [26]. Features were derived from data contained in the voxels of the segmented structures and can be grouped into different categories. First-order features were derived from the histogram of voxel intensities (SUVmean, SUVmax, skewness, kurtosis, etc.). Second-order textural features were based on matrices that contained information about the regional spatial arrangement of the voxels such as their homogeneity, contrast, and coarseness simulating the human perception of the image. Higher-order features such as Grey-level run length features focused on local collinear voxels with the same grey level.

The TA features analyzed are reported in Table 1 of reference [26]. A detailed description of the features is also reported in the supplemental material of the paper of Aerts et al. [27]. Images were digitized in 64 digitization bins according to the minimum and maximum values in the segmented volume. Tumor volumes smaller than 2.6 ml were excluded from the statistics due to insufficient number of voxels to perform texture analysis. This choice was based on previous evaluation about feature stability on NEMA IEC quality assurance phantom (2.6 ml corresponds to the volume of the 3rd sphere of the NEMA IEC phantom in the increasing order) [24, 28].

### 2.5. Statistical Analysis

First, associations of demographic and clinical characteristics of patients, such as sex, age, clinical stage (III vs IV), and tumor site (oropharynx vs other sites), with local control, progression-free survival (PFS), and overall survival (OS) were tested with univariate and multivariate analysis (Cox proportional hazard model). In particular, given the high heterogeneity of the population, we decided to create two tumor groups that are oropharynx and other sites (hypopharynx, larynx, oral cavity, and nasopharynx) in order to test its effect on survival. Furthermore, correlation between clinical parameters and predictive imaging biomarkers was investigated through spearman *ρ*.

Texture parameters were extracted and then compared in patients with local control against patients with local failure. To select both clinical and imaging variables that are more related to clinical outcomes, we used a “least absolute shrinkage and selection operator” (LASSO) method. LASSO can “shrink” the effect of unimportant features and can set their effects to zero together with removing redundancy among the features.

Given the iterative nature of LASSO algorithm, it was run one thousand times in order to have a statistics of the most descriptive clinical and imaging features. The most occurring features (more than 500 times over 1000 runs) were selected to build the final image biomarker-based multivariate Cox regression analysis. An internal 10-fold cross-validation algorithm was applied for validation. Finally, hazard ratios (HR) and confidence intervals (CI) were calculated. Discrimination, reflecting a correct ordering of the relative predictions for individuals, and the model's ability to distinguish patients with local control against patients with local failure, were determined by the Harrell's concordance-index (*c*-index) with R statistical package-based code (https://www.r-project.org/). *c*-index is an extension of ROC curves for multivariable models with 0.5 value indicating random discrimination and 1 perfect discrimination capabilities. The difference in prognostic value between the Cox model developed with or without imaging biomarkers was evaluated based on the comparison between Harrell's concordance indexes. Finally, Kaplan–Meier curves have been calculated in order to show selected predictor stratification capabilities.

## 3. Results

At a median follow-up of 38 months (range 24–848 months), one hundred patients (79%) were alive, 13 died due to disease progression, and 12 due to other causes. Four patients were lost at follow-up.

Eighteen early-stage patients were excluded due to the small tumor volume (<2.6 ml) as textural features becomes unstable or even undefined due to small number of voxels involved; 13 patients due to technical problems regarding PET/CT acquisition (different acquisition, reconstruction protocols, or missing uptake data). The final number of evaluated patients for statistical analysis was 90. Of these 90 patients, 62 showed no evidence of disease (NED) at last follow-up; 28 patients (30%) had recurrent disease (RD), of which 21 patients developed locoregional recurrences and 7 isolated distant metastases.

The pattern of recurrence was analyzed in 21 patients who had locoregional relapse: “in-field” (*if >80% of the tumor recurrence resided within the prescription 95% isodose surface*) in 20 patients (95%), at RT field margin (*if 20–80% of the lesion was inside the 95% isodose surface*) in no patients (0%), and “out-field” in one patient (5%).

The estimated 2- and 5-year OS rates were 86% (95% CI 79–92%) and 68 % (95% CI 58–78%), respectively. The corresponding 2- and 5-year PFS rates were 70% (95% CI 58–77%) and 66% (95% CI 55–75%), respectively.

Results of univariate analysis are showed in Table 2. No standard clinical and demographics parameters showed a statistically significant correlation with PFS. Gender and age showed significant correlation for OS. In particular, gender seems to have a strong impact on survival in our dataset (HR = 8.316). Clinical stage was correlated with both PFS and OS with an HR of 1.76 and 1.92, respectively, without any statistical significance.

At multivariate analysis, shown in Table 3(A), no standard clinical parameters were correlated with PFS, and the *c*-index resulted in 0.65 in the ability of prediction of the model, without statistical significance. Regarding OS, the multivariable Cox model showed a statistically significant correlation with both gender and age with a *c*-index of 0.73 as can be seen in Table 4(A). It is noticeable that in any Cox regression, tumor site seems to have no effects on survival prediction for our population. We can then state that this parameter will not influence our further models with imaging biomarkers.

The LASSO algorithm revealed that four parameters, including run percentage, low-intensity long-run emphasis (LILRE), coarseness, and code similarity, showed significant differences between local failure and local control groups, and these parameters were selected, together with two clinical variables (age and stage), to build a multivariate Cox regression for PFS. No significant correlation exists between the analyzed parameters as spearman rho coefficient is always below 0.6.

**Figure 1:**
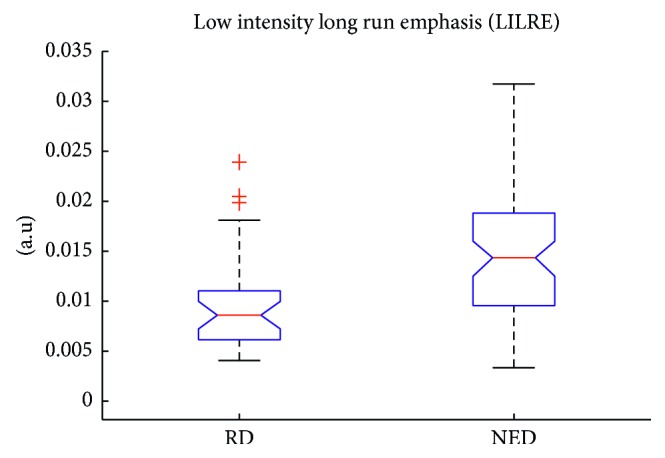
Low-intensity long-run emphasis values calculated on different populations in arbitrary unit of measure: recurrent disease (RD) and no evidence of disease (NED) patients.

**Figure 2:**
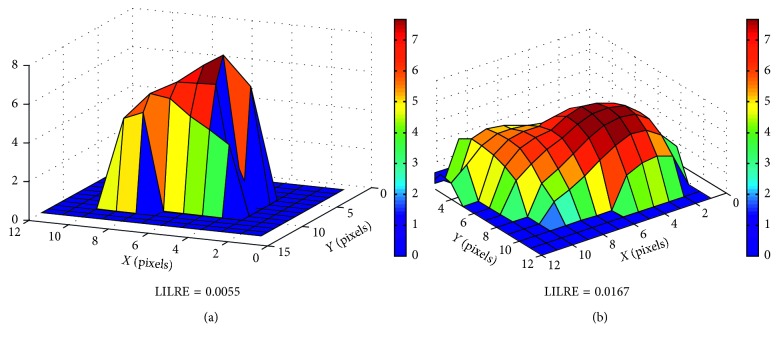
Detail of the uptake of 2D slices centered in the middle of 2 tumor VOIs. On the left, the slice is taken from a RD patient and on the right from a NED patient. It can be appreciated that a lower value in LILRE produces a more speculated uptake in the tumor, whereas in NED patient, the uptake is more Gaussian like. (a) RD patient. (b) NED patient.

**Figure 3:**
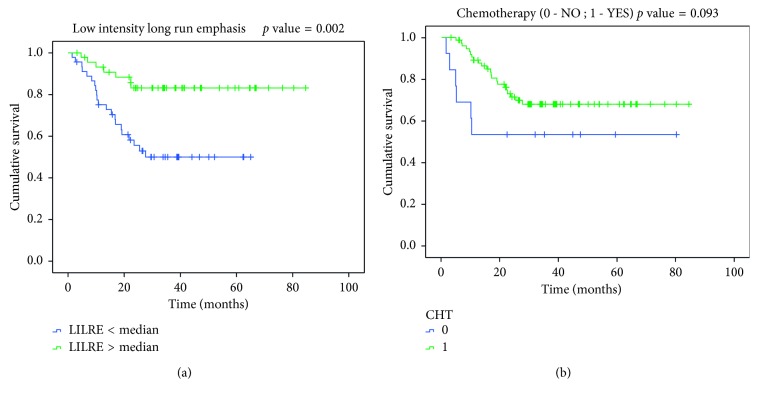
Kaplan–Meier survival curves for the two statistically significant predictors emerged from the multivariate Cox regression. In (a), the median value of low-intensity long-run emphasis feature is employed to split the curves of the patients, whereas in (b), the split is performed by using chemotherapy usage (Yes or No).

In Table 3(B) the resulting model for PFS is shown; the Harrel *c*-index is of 0.76 with a statistical significance of *p* < 0.01 at cross-validation. Also the comparison of the two *c*-indexes underlined a difference between the two models with statistical significance and *p* < 0.01. In particular LILRE, which is the strongest predictor in the model, is associated with the presence of long string of low grey level pixel in the tumor contours. In Figure 1 the values of the feature in RD and NED patients are shown. An evident difference in median values appears between the populations. The Wilcoxon Test scored a *p* value of 0.006 between RD and NED patients. Furthermore, in Figure 2, the difference in a 2D render of the morphology of the 2 populations (representative patients) is revealed. In RD patients, it is evident how the borders of the tumors are sharper in comparison to NED patients. Finally, in Figure 3(a), Kaplan–Meier survival curves for LILRE, where survival curves are split by the median value of LILRE, and in Figure 3(b), the effect of the use of chemotherapy (CHT) are shown. Pearson correlation coefficient *ρ* calculated between LILRE and the other clinical variables was always below 0.2, confirming the independence of this imaging biomarker.

By adding imaging biomarkers and processing variables in the same way of PFS dataset, we end up with the model shown in Table 4(B). This model, which includes five variables (gender, age, staging, low-intensity large-zone emphasis, and SUL peak), achieves a *c*-index of 0.76, but it is not significantly different from the model with only the clinical variables.

## 4. Discussion

To date, there is increased evidence suggesting that genomic heterogeneity of aggressive tumors could translate into intratumoral spatial heterogeneity, which can be represented on anatomical and functional scales [18, 27, 29, 30].

This is the central idea that underlies radiomics, in which large amounts of information through advanced quantitative analysis of radiological images are used as noninvasive means to characterize intratumoral heterogeneity and to identify important prognostic features of cancer [27, 31–34]. Nowadays, medical imaging plays a central role in the investigation of intratumoural heterogeneity, as radiological images are acquired as routine practice for almost every patient with cancer. Medical images such as ^18^F-FDG PET/CT and CT are minimally invasive and include an immense source of potential data for decoding tumour phenotypes.

Texture feature-based analysis of clinical outcomes is actively being investigated as a prognostic tool in radiation oncology in order to identify potentially predictive radiomic biomarkers for either clinical outcomes (local control and/or treatment toxicity) or distinguishing local recurrence from radiation-induced injury. Some studies have also been performed to identify radiomic signatures for HNSCC. Aerts et al. analyzed radiomic values of CTs from 1,019 patients suffering from non-small-cell lung cancer or head and neck cancer in relation to prognostic features and showed that combining radiomic signature with TNM staging improved the predictive power in all groups of patients [27]. Parmar et al. investigated 440 radiomic features extracted from the CTs of 878 lung cancers and HNSCC patients and showed that, at multivariate analysis, the radiomic feature clusters highly correlate with tumor stage and moderately correlate with HPV status [34]. In an additional study, the same group established a reliable machine-learning method for the prediction of OS in HNSCC patients based on CT scan-derived radiomic features. Here, training was performed on 440 radiomic features among a cohort of 101 HNSCC patients, while another 95 patients served as the validation cohort. In this study, authors showed that machine-learning methods had a high predictive power with a good stability and believed that this technique could improve the application of radiomics in cancer [35]. These 3 publications all suggest a relevance of radiomics in HNSCC and a potential future role in HNSCC classification and treatment to improve clinical decision-making. Recently, it was reported that a 4-feature-based radiomic signature showed strong correlation with survival and distant metastasis in oropharyngeal squamous cell carcinoma [36]. The concordance index (CI) in training cohorts and validation cohorts were ranging from 0.55 to 0.69.

Vallières et al. recently investigated the possibility of constructing a prediction model integrating clinical information with radiomics analysis, using advanced machine learning, in patients with HNSCC treated with chemoradiation. 1,615 different radiomic features were extracted from PET and CT pretreatment images of 300 patients, and the results demonstrated the potential of radiomics for assessing the risk of specific tumor outcomes using multiple stratification groups [10].

In this study, we analyzed a cohort of 90 patients with diagnosis of HNSCC using a multivariate Cox model comparing features based on clinical variables and imaging biomarkers. Univariate analysis showed how gender and age are important predictors for OS. The effect of gender on overall survival is a known phenomenon, as shown also recently in the large European multicenter study [37]. This trial reported that a significant effect on OPC survival was apparent for female sex (aHR: 0.50; 95% CI: 0.29–0.85).

Models including imaging biomarkers were always superior to those with only clinical variables, even if only in the case of PFS, we had a statistically significant difference. The multivariable model developed with clinical variables alone scored a *c*-index of 0.65, whereas including imaging biomarkers, a *c*-index of 0.76 was reached in predicting patient outcome as PFS. Furthermore, the comparison of the *c*-index underlined the significance of the difference between the two models. This improvement in patient stratification can be addressed to the introduction of the textural feature LILRE which is strongly associated with patient disease recurrence, as shown in the box plot in Figure 1 and in Kaplan–Meier curves in Figure 3(a) employing the median value of LILRE over the entire population as split value. It is also interesting to notice that this feature can be associated to morphological properties of the image itself. These findings underline the importance of further studies to validate the use of imaging biomarkers to improve patients' stratification and prognostic accuracy.

Our study has several limitations, and should be considered as a preliminary experience with need for possible methodological and technical refinements, beyond independent external validation. It is a retrospective study with a relatively small population. The characteristics of enrolled patients were heterogeneous, as well as disease sites, and the selection of treatment might be biased, so that the significance of features can be underestimated.

Despite this, it seems that heterogeneity is not influencing the results as no standard Cox regression models gives any statistically significant association with survival in this cohort. Furthermore, no correlation was found between our main predictor (LILRE) and tumor site. Additionally, HPV status was not determined in all patients, and it has not been considered as a predictive factor. Although especially in oropharyngeal cancer infection with HPV has a predictive value for treatment response [4, 38], no different radiomic features were identified between HPV-positive and HPV-negative patients.

In the other two analyses, statistically significant differences in some texture features between HPV+ and HPV− HNSCC were found, even if a single radiomic feature may not have enough predictive power [39, 40].

Despite the limitations of this study, our results appear to be promising. In order to confirm these findings, external validation will be performed, and for the case of oropharynx, tumor HPV infection data will be included.

## 5. Conclusion

We compared a clinical variables alone statistical model with another one which includes imaging biomarkers. Superiority of the imaging biomarkers model was proven in the case of PFS. Further studies will be aimed at confirming our findings with an external dataset and to demonstrate the superiority of models comprising imaging biomarkers also for overall survival.

## Figures and Tables

**Table 1:** Patients' clinical and demographic data.

	Number of patients (%)
*N*	90
*Mean age (range)*	60 (22–87)
≥60 years	52 (58%)
<60 years	38 (42%)

*Gender*	
Male	68 (75%)
Female	22 (15%)

*Primary site*	
Oral cavity	4 (4%)
Oropharynx	49 (55%)
Hypopharynx	10 (10%)
Nasopharynx	13 (15%)
Larynx	14 (16%)

*Clinical T stage*	
T1	15 (17%)
T2	30 (35%)
T3	29 (33%)
T4	13 (15%)

*Clinical N stage*	
N0	5 (5%)
N1	21 (24%)
N2	59 (66%)
N3	5 (5%)

*Clinical stage*	
III	32 (35%)
IV	58 (65%)

*Patient cohort after follow-up (PFS)*	
NED	62 (69%)
RD	28 (31%)

*Patient cohort after follow-up (OS)*	
Alive	65 (72%)
Dead	25 (28%)

**Table 2:** Univariate model of patients' clinical data with progression-free survival (PFS) and overall survival (OS). The number of patients is 90 for both PFS and OS, whereas the number of events is 28 and 25, respectively.

	*p* value	HR	95% CI for HR	
			Lower	Upper
*Univariate Cox regression for PFS*				
Gender (F vs M)	0.698	1.196	0.485	2.951
Age	0.099	1.027	0.995	1.060
Stage (III vs IV)	0.197	1.758	0.747	4.137
Tumor site (oroph vs other)	0.914	1.043	0.488	2.227
CHT (No vs Yes)	0.101	0.470	0.190	1.159

*Univariate Cox regression for OS*				
Gender (F vs M)	0.038	8.495	1.149	62.820
Age	0.004	1.052	1.016	1.089
Stage (III vs IV)	0.169	2.010	0.797	5.070
Tumor site (oropharynx vs others)	0.772	0.886	0.391	2.009
CHT (No vs Yes)	0.838	0.895	0.307	2.608

**Table 3:** Multivariate analysis of the patients' dataset for PFS without (A) and including (B) imaging biomarkers. Harrel *c*-indexes of the models, which score their prognostic power, are 0.65 and 0.76, respectively. The two *c*-indexes are significantly different with a *p* value of 0.01.

	*p* value	HR	95% CI for HR	
			Lower	Upper
*(A)*				
Gender (F vs M)	0.713	1.191	0.469	3.026
Age	0.426	1.014	0.979	1.050
Stage (III vs IV)	0.160	1.894	0.777	4.617
Tumor site (oropharynx vs others)	0.902	0.953	0.445	2.042
CHT (No vs Yes)	0.176	0.489	0.173	1.378

*(B)*				
Age	0.254	1.02*E* + 00	9.86*E* **−** 01	1.06*E* + 00
Stage (III vs IV)	0.440	1.43*E* + 00	5.77*E* **−** 01	3.55*E* + 00
*CHT (No vs Yes)*	**0.012**	**2.03E** **−** **01**	**5.84E** **−** **02**	**7.06E** **−** **01**
Run percentage	0.176	1.97*E* **−** 01	1.87*E* **−** 02	2.07*E* + 00
*LILRE*	**<0.001**	**1.41E** **−** **83**	**4.05E** **−** **128**	**4.90E** **−** **39**
Coarseness	0.970	4.12*E* **−** 01	2.32*E* **−** 21	7.33*E* + 19
Code similarity	0.129	4.27*E* **−** 23	5.77*E* **−** 52	3.16*E* + 06

**Table 4:** Multivariate analysis of the patients dataset for OS without (A) and including (B) imaging biomarkers.

	*p* value	HR	95% CI for HR	
			Lower	Upper
*(A)*				
Gender (F vs M)	**0.042**	**8.036**	**1.082**	**59.770**
Age	**0.017**	**1.057**	**1.012**	**1.109**
Stage (III vs IV)	0.262	1.734	0.663	4.535
OvsL	0.895	0.937	0.395	2.029
Novscht	0.317	1.916	0.521	7.051

*(B)*				
Gender (F vs M)	0.090	5.75*E* + 00	7.90*E* **−** 01	4.81*E* + 01
Age	**0.011**	**1.05E** **+** **00**	**1.01E** **+** **00**	**1.09E** **+** **00**
Stage (III vs IV)	0.341	1.59*E* + 00	5.82*E* **−** 01	3.83*E* + 00
LILRE	0.635	7.08*E* **−** 05	9.72*E* **−** 25	8.35*E* + 9
SUL peak	0.068	1.10*E* + 00	1.009*E* + 00	1.192*E* + 00

## Data Availability

The data used to support the findings of this study are available from the corresponding author upon request.
